# Changes in beta cell function occur in prediabetes and early disease in the *Lepr*^*db*^ mouse model of diabetes

**DOI:** 10.1007/s00125-016-3942-3

**Published:** 2016-04-05

**Authors:** Oanh H. Do, Jenny E. Gunton, Herbert Y. Gaisano, Peter Thorn

**Affiliations:** School of Biomedical Sciences, University of Queensland, St Lucia, Brisbane, QLD 4072 Australia; Charles Perkins Centre, University of Sydney, John Hopkins Drive, Camperdown, Sydney, NSW 2050 Australia; Westmead Hospital, Sydney, NSW 2145 Australia; Westmead Institute for Medical Research, PO Box 412, Westmead, Sydney, NSW 2145 Australia; School of Medicine, University of Toronto, Toronto, ON M5S 1A8 Canada

**Keywords:** Beta cell, Compound exocytosis, Exocytosis, Insulin granules, Islets, Prediabetes, Progression

## Abstract

**Aims/hypothesis:**

Type 2 diabetes is a progressive disease that increases morbidity and the risk of premature death. Glucose dysregulation, such as elevated fasting blood glucose, is observed prior to diabetes onset. A decline in beta cell insulin secretion contributes to the later stages of diabetes, but it is not known what, if any, functional beta cell changes occur in prediabetes and early disease.

**Methods:**

The *Lepr*^*db*^ mouse (age 13–18 weeks) was used as a model of type 2 diabetes and a two-photon granule fusion assay was used to characterise the secretory response of pancreatic beta cells.

**Results:**

We identified a prediabetic state in *db*/*db* mice where the animals responded normally to a glucose challenge but have elevated fasting blood glucose. Isolated islets from prediabetic animals secreted more and were bigger. Insulin secretion, normalised to insulin content, was similar to wild type but basal insulin secretion was elevated. There was increased glucose-induced granule fusion with a high prevalence of granule–granule fusion. The glucose-induced calcium response was not changed but there was altered expression of the exocytic machinery. *db/db* animals at the next stage of disease had overt glucose intolerance. Isolated islets from these animals had reduced insulin secretion, reduced glucose-induced granule fusion events and decreased calcium responses to glucose.

**Conclusions/interpretation:**

Beta cell function is altered in prediabetes and there are further changes in the progression to early disease.

**Electronic supplementary material:**

The online version of this article (doi:10.1007/s00125-016-3942-3) contains peer-reviewed but unedited supplementary material, which is available to authorised users.

## Introduction

Type 2 diabetes is a chronic, progressive disease characterised by loss of glucose homeostasis and leading to morbidity and premature mortality [1–4]. A key feature of the later disease stages is the failure of insulin secretion from beta cells to meet the demand of increased peripheral insulin resistance [1, 5]. However, in early disease there can be an increase in insulin secretion that might be explained by an increase in beta cell mass; nevertheless, it is unknown whether beta cell function also changes. Here, we have used the *Lepr*^*db*^ mouse model of diabetes to investigate beta cell function in early disease.

Prior to overt type 2 diabetes, a prediabetic state can be identified by impaired fasting glucose (IFG) or impaired glucose tolerance (IGT) after a glucose tolerance test (GTT). A study of prediabetic individuals (identified by IFG) showed increased insulin secretion in response to an OGTT [6]. Some studies have also shown that individuals with IGT have enhanced insulin secretion [6, 7], but it is not known whether this is due to upregulation of beta cell secretion or an increased number of beta cells [3].

There is indirect evidence that compensatory increases in beta cell number can occur in obese humans [8, 9] and in animal models such as prediabetic Zucker diabetic fatty rats [10], TALLYHO diabetic mice [11], *ob*/*ob* mice [12] and animals fed a high-fat diet [13–15].

There is no direct evidence that beta cell function changes in prediabetes or early disease. However, it is known to occur in other contexts. For example beta cell secretory function is enhanced in pregnancy to cope with the increased demand from the developing fetus [16]. Moreover, in the *ob*/*ob* model of obesity, in addition to increased numbers of beta cells, there is increased secretion per cell [12].

In this paper, we describe experiments using the *Lepr*^*db*^ mouse model of type 2 diabetes [17] aimed at understanding beta cell function during prediabetes and early disease. Our results show beta cells do undergo functional changes during prediabetes and their exocytic capacity is upregulated in response to glucose. We conclude that pathological changes are already occurring in beta cells during prediabetes.

## Methods

### Glucose tolerance test

We performed i.p. glucose tolerance tests (1 g/kg body weight) for wild-type (WT, +/+) and all *db/db* mice (see electronic supplementary material [ESM] Methods for details).

### Cell preparation

Mice of both sexes and aged 13–18 weeks were used, except where otherwise stated (BKS.Cg-*Dock7m*+/+*Lepr*^*db*^/J, The Jackson Laboratory, Bar Harbor, ME, USA) were humanely killed according to local animal ethics procedures (approved by the University of Queensland Anatomical Biosciences Ethics Committee). Islets were prepared by enzymatic digestion of the pancreas from WT, stage 1 and stage 2 *db*/*db* mice for experiments (except the insulin assay, which was performed in WT and *db*/*db* mice of all stages; see ESM Methods for details).

### Experimental solution

Two-photon imaging, insulin assays and calcium measurements were performed in a sodium-rich extracellular solution (see ESM Methods for details).

### Two-photon imaging

Isolated islets were first cultured for 2–3 days and then prior to imaging were bathed in an extracellular solution containing 3 mmol/l glucose for 30 min (37°C, 95%/5% air/CO_2_). Islets were then transferred to an extracellular solution containing 15 mmol/l glucose and 0.8 mmol/l sulforhodamine B (SRB). Two-photon imaging was performed at 34°C, with exocytic events recorded as entry of the SRB extracellular dye (excitation 950 nm, detection 550–650 nm) into each fused granule (see ESM Methods for details).

### Insulin assay

Homogeneous time resolved fluorescence with the HTRF Insulin Kit (no. 62INSPEB, Cisbio Bioassays, Brisbane, QLD, Australia) was used to measure islet insulin secretion (see ESM Methods for details).

### Calcium measurement

For intracellular calcium measurement, we used the ratiometric calcium indicator Fura2-AM (see ESM Methods for details).

### Immunofluorescence

Freshly isolated islets were fixed in 4% paraformaldehyde, permeabilised with Triton X-100 and blocked with donkey serum containing BSA for 1 h prior to incubation with primary antibodies (see ESM Methods for details).

### Quantitative real-time PCR

Total RNA was isolated from islets using RNeasy plus Micro kit (Qiagen). cDNA was synthesised from 100 ng total RNA using the SuperScript III reverse transcriptase (Invitrogen, Mt Waverley, VIC, Australia; see ESM Methods for details).

### Serial block-face scanning electron microscopy

Islets cultured for 2 days were stimulated with 15 mmol/l glucose for 7 min and then fixed with 2.5% glutaraldehyde and processing for imaging (see ESM Methods for details).

### Statistical analyses

All numerical data are presented as the mean ± SEM. Statistical analysis was performed using Microsoft Excel 2010 (Microsoft Corporation, Redmond, WA, USA) and GraphPad Prism version 6 (La Jolla, CA, USA). Datasets containing just two groups were subjected to a two-tailed, unpaired Student’s *t* test with statistical significance identified at *p* < 0.05. Islets from at least three animals were used in each experiment. Statistical significance is indicated in the figures as *p < 0.05, **p < 0.01, and ***p < 0.001.

## Results

**Fig. 1:**
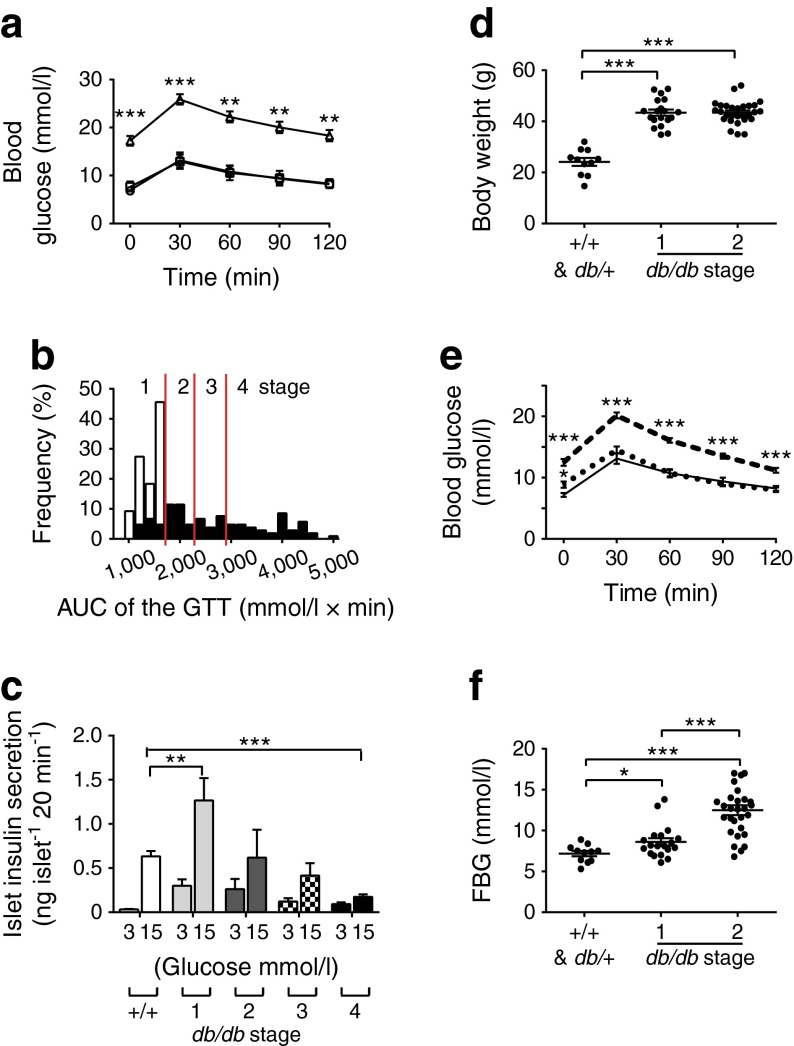
Classification of *db/db* animals into separate stages of disease. (**a**) The overall mean GTT was significantly impaired in *db/db* (triangles) compared with WT (+/+; circles) and +/− mice (squares). (**b**) *db/db* mice (black bars) are classified into four stages based on the AUC for the GTT: stage 1, <1,600 mmol/l × min; stage 2, 1,600–2,200 mmol/l × min; stage 3, 2,200–2,800 mmol/l × min; stage 4, >2,800 mmol/l × min. White bars, WT. (**c**) Glucose-induced islet insulin secretion is increased at stage 1 and reduced for the later stages of disease. (**d**) Stage 1 animals are significantly heavier than WT (+/+) animals. (**e**) GTT is comparable in stage 1 *db/db* (dotted line) and WT (+/+) islets (solid line) but significantly increases in stage 2 *db/db* islets (dashed line). (**f**) Stage 1 *db/db* mice have a small but significant increase in FBG. *p < 0.05, **p < 0.01, ***p < 0.001 vs WT

The *Lepr*^*db*^ spontaneous mutant (BKS.Cg-*Dock7m*+/+*Lepr*^*db*^/J) is a recognised mouse model for type 2 diabetes [17]. The *db/db* animals gained weight and developed disease, as assessed in 13–18-week-old animals using GTTs (Fig. 1a; seven WT, four *db*/+, 105 *db/db* mice). As in human disease [18], there is great variability in phenotype among individual animals. In *db/db* animals, the AUC (derived from the GTTs) ranged from normal to a fivefold increase (Fig. 1b). We categorised the AUC into four stages of severity, stage 1 (compensation) to stage 4 (severe disease), analogous to those of human disease [3]. Performing GTTs before each animal was sacrificed thus enabled us to classify the severity of their disease.

We next tested islet function to determine whether insulin secretion changed according to the stage of disease severity. Isolated islets were stimulated for 20 min with 3 or 15 mmol/l glucose. At stage 1, *db/db* islets showed a significant increase in insulin secretion at both 3 and 15 mmol/l glucose compared with WT (Fig. 1c; 24 WT and 25 *db/db* mice). At stage 2, insulin secretion decreased to levels resembling those of WT islets; at stage 3, there was a further decrease in insulin secretion; and at stage 4, insulin secretion was significantly lower than in WT islets (Fig. 1c).

We used this classification to subdivide the animals’ phenotype. At stage 1, *db/db* animals were heavier than WT (Fig. 1d), with no significant difference in the GTT (Fig. 1e) and a small, but significant, increase in fasting blood glucose (FBG) (Fig. 1f). By stage 2, the body weight, GTT and FBG of *db/db* animals all differed significantly from those of WT animals and were indicative of frank diabetes (Fig. 1d–f). These changes approximate to the progression of human type 2 diabetes.

### Islet size and insulin content increase in prediabetes

**Fig. 2:**
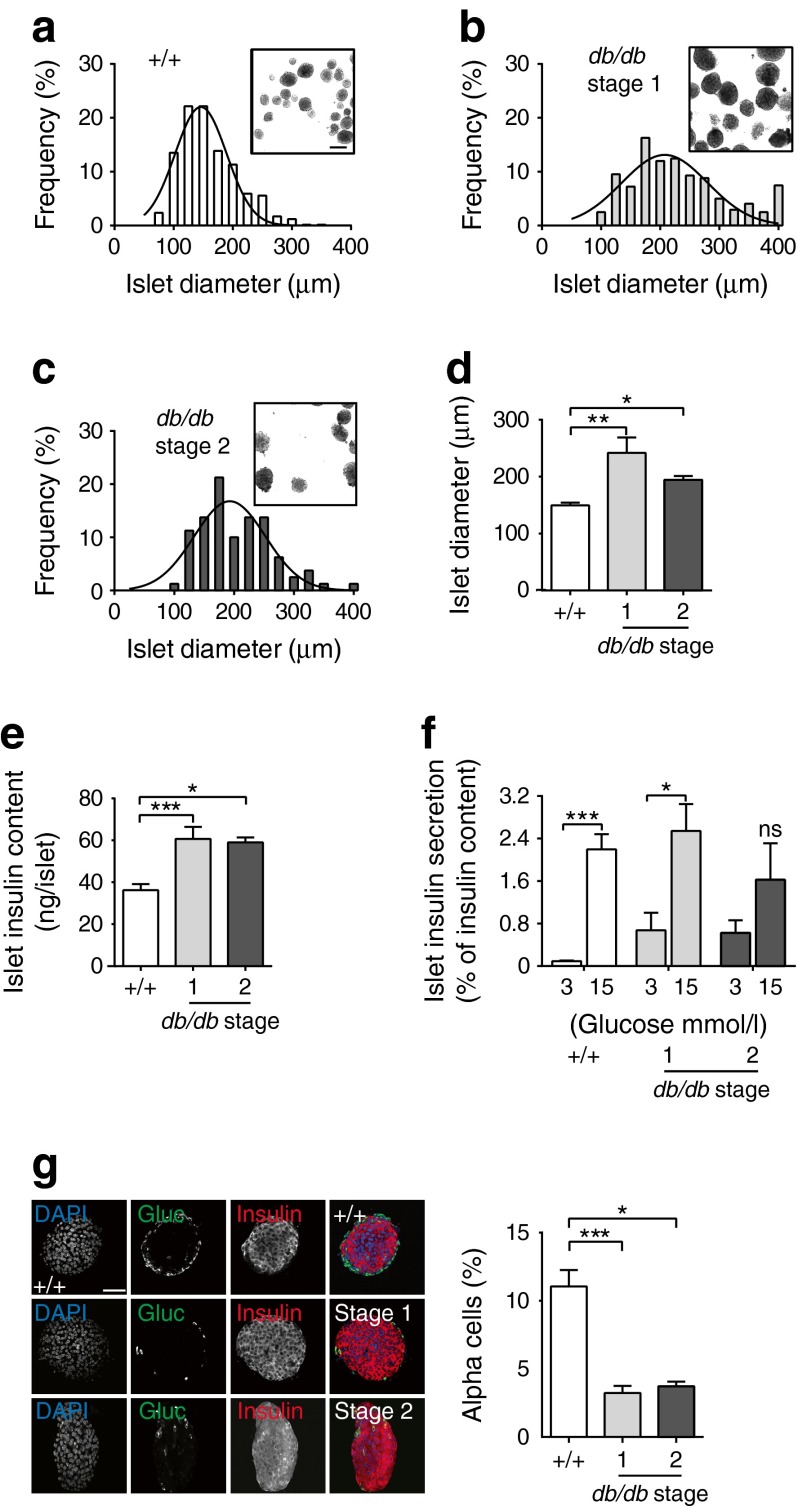
Islet morphology changes in *db/db* islets. Stage 1 and stage 2 *db/db* islets have (**a–d**) a larger size (scale bar 200 μm) and (**e**) increased islet insulin content compared with WT (+/+) islets. (**f**) Insulin secretion, expressed as percentage insulin content for WT (+/+) and stage 1 and stage 2 *db/db* islets in response to 3 and 15 mmol/l glucose. (**g**) Immunostaining shows a selective expansion in beta cells compared with alpha cells in stage 1 and 2 *db/db* islets compared with WT. Scale bar 50 μm. *p < 0.05; **p < 0.01; ***p < 0.001. Gluc, glucagon

The basis of increased insulin secretion during the prediabetic phase could be an increase in islet size. Consistent with this, islets isolated from WT, stage 1, and stage 2 *db/db* animals showed an increase in islet size (Fig. 2a–d) and insulin content (Fig. 2e). The islet composition also changed: there was a decreased relative percentage of alpha cells (Fig. 2g). When normalised to the islet content, the increase in basal secretion persisted but the increase in glucose-induced secretion (Fig. 1c) was no longer apparent (Fig. 2f). We therefore expected to see little change in secretory output per cell.

### Insulin secretion during stage 1 shows upregulation of the exocytic capacity

To study secretory function within individual beta cells in intact islets, we used a two-photon, granule fusion assay that we previously used to characterise the fusion of individual insulin granules from beta cells within intact islets [19]. When recording from the islet core, where most cells are beta cells (see Fig. 2g), this method detects the fusion of granules that are the same size as insulin granules, colocalise with insulin and, in terms of fusion numbers, fully account for insulin secretion from the islets [19].

**Fig. 3:**
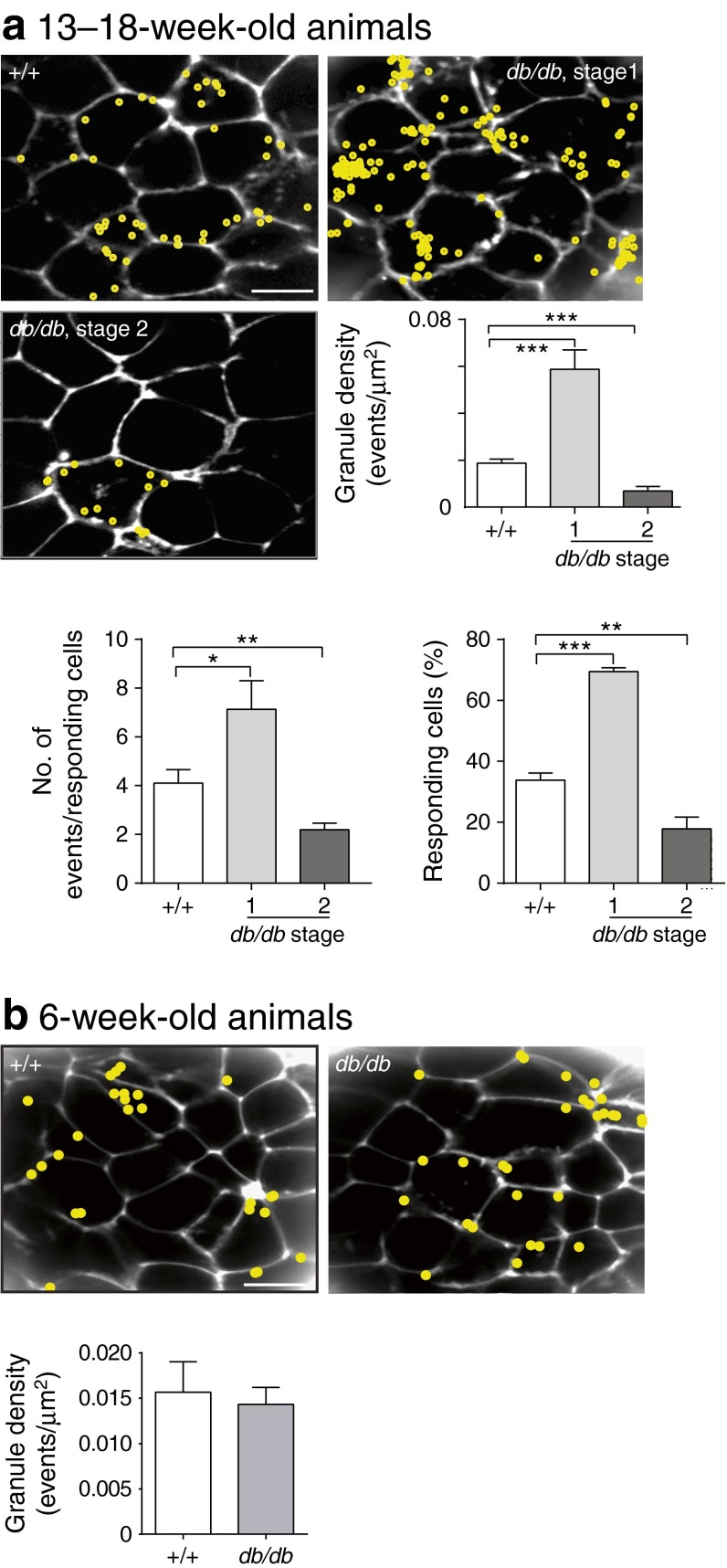
Glucose-induced insulin granule fusion is substantially altered in *db/db* islets. (**a**) A two-photon granule fusion assay identified the sites of granule fusion events (yellow circles) over a 20 min period in response to 15 mmol/l glucose. Compared with WT (+/+), many more fusion events are seen in stage 1 *db/db* islets, with greater spatial clustering of fusion events. In contrast, stage 2 *db/db* islets show a dramatic decrease in fusion events. Histograms show changes in granule exocytosis in terms of the numbers of responding cells and responses per cell within the two-photon volume. (**b**) At 6 weeks of age, the number of glucose-induced exocytic events is comparable with that of age-matched WT (+/+) islets. Scale bars 10 μm. *p < 0.05, **p < 0.01, ***p < 0.001 vs WT

Islets were stimulated with 15 mmol/l glucose for 20 min. As each granule fuses with the cell membrane, extracellular dye enters the granule, thus enabling the precise spatial and temporal localisation of the fusion event (Fig. 3a). Compared with WT islets, there are more granule fusion events in stage 1 islets, demonstrating that functional changes are occurring in beta cells. At stage 2, there are fewer granule–granule fusion events compared with WT. This was expected because these animals have overt diabetes, and has been previously reported by us [20].

The increased exocytic response at stage 1 reflects both an apparent increase in the numbers of cells responding to glucose and an increase in the number of fusing granules per cell (Fig. 3a; nine WT and 21 *db/db* mice). This shows that individual beta cells have become more responsive to a glucose stimulus.

These data were all obtained from 13- to 18-week-old animals. To test whether the *db/db* animals were born with the increased insulin granule exocytosis seen at stage 1 or progressed to this point, we studied 6-week-old *db/db* animals classified as stage 1 by their GTT responses. These animals had not yet developed abnormal FBG; in addition, their islet diameter, insulin content and insulin secretion were indistinguishable from those of WT animals (Table 1). After glucose stimulation, the number of exocytic events in 6-week-old *db/db* animals was not different from that of age-matched WT animals (Fig. 3b). These data indicate that the enhanced granule–granule fusion seen in stage 1 animals aged 13–18 weeks is an adaptive response to disease progression.

**Table 1:** Phenotype of 6-week-old WT and stage 1 *db/db* mice

Variable	+/+	*db*/*db*	*p* value^a^
AUC (mmol l^−1^ min^−1^)	916 ± 43	1,076 ± 108	0.24
FBG (mmol/l)	6.3 ± 0.5	7.6 ± 1.0	0.34
Islet diameter (μm)	145 ± 7	132 ± 11	0.41
Islet insulin content (ng/islet)	40.2 ± 10.3	43.2 ± 8.7	0.83
Islet insulin secretion^b^ (% of insulin content)	1.23 ± 0.43	1.43 ± 0.41	0.75

Data are expressed as the mean ± SEM. *n* = 3 for all values

^a^Student’s *t* test

^b^In response to 15 mmol/l glucose

**Fig. 4:**
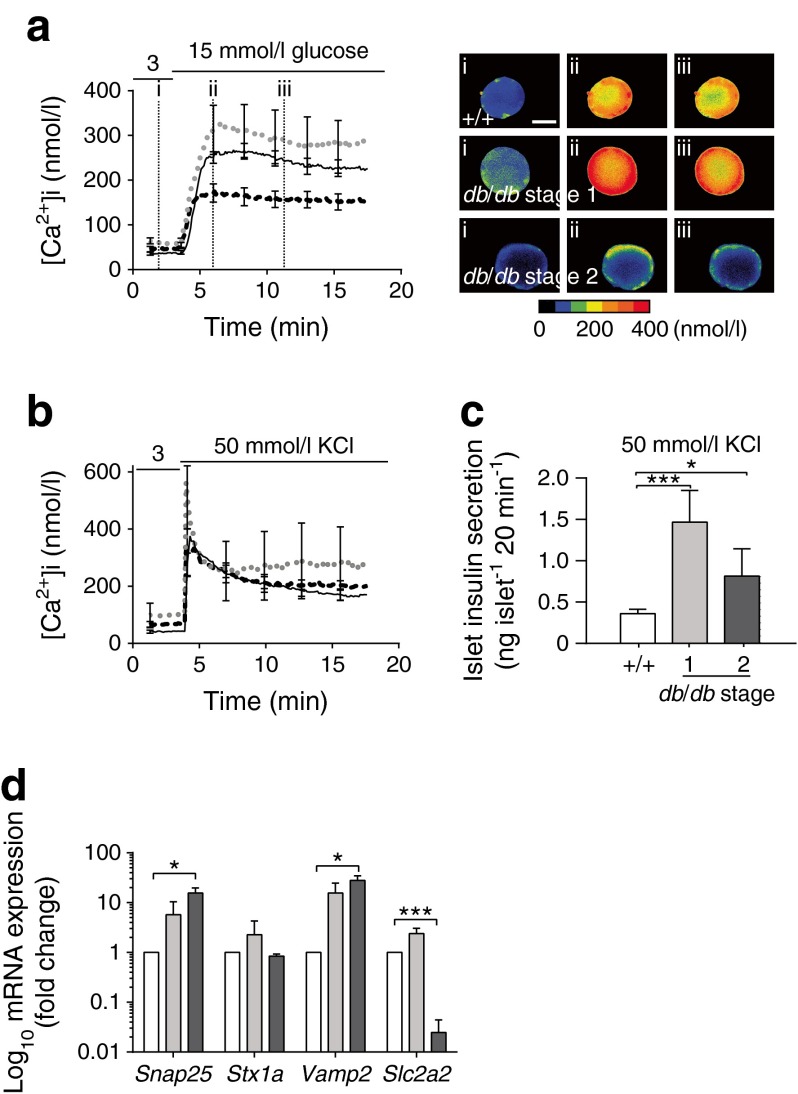
Characterisation of beta cell changes in stage 1 and 2 *db/db* islets. (**a**) Glucose-induced islet calcium responses are comparable between WT (solid line) and *db/db* stage 1 islets (grey dotted line) but reduced in *db/db* stage 2 islets (black dashed line). Scale bar 50 μm. (**b**) Potassium-induced islet calcium responses are comparable among WT (solid line), *db/db* stage 1 (grey dotted line) and *db/db* stage 2 (black dashed line) islets. (**c**) Responses to 50 mmol/l KCl show enhanced insulin secretion at stage 1 and 2 in *db/db* compared with WT (+/+) beta cells. (**d**) qPCR shows elevations in SNARE mRNA expression at stage 1 (light grey bars) and 2 (dark grey bars) but a reduction in *Slc2a2* mRNA at stage 2 in *db/db* compared with WT (white bars) beta cells. *p < 0.05, ***p < 0.001 vs WT

These changes in insulin granule fusion could be due to changes in the exocytic machinery or to upstream changes, such as altered calcium responses that are triggered by glucose. Using Fura-2 loading, we showed higher resting calcium levels in stage 1 *db/db* than in WT islets (64 ± 6.8 nmol/l in stage 1 vs 35.5 ± 3.4 nmol/l in WT islets, *p <* 0.01, Student’s *t* test) but no significant difference in the peak glucose-induced calcium response (Fig. 4a). In contrast, stage 2 islets showed reduced calcium responses (Fig. 4a). This result indicates that the increase in granule–granule fusion at stage 1 is not due to an increased calcium response and instead suggests specific upregulation of the granule fusion process. This conclusion is supported by experiments using high extracellular potassium as a stimulus that directly depolarises cells and bypasses glucose metabolism. The high potassium-induced calcium response was similar in stage 1 *db/db* as in WT islets (Fig. 4b) but there was greater insulin secretion (Fig. 4c). This evidence for an enhanced exocytic capacity in stage 1 *db/db* islets is supported by quantitative real-time PCR (qPCR) data, which show upregulation of the mRNAs for soluble *N*-ethylmaleimide-sensitive factor attachment protein receptors (SNAREs), *Snap25* and *Vamp2*, although the *Stx1a* mRNA level does not change significantly (Fig. 4d).

Our data also indicate the mechanism responsible for decreased insulin secretion at stage 2. Stage 2 islets showed continued upregulation of the mRNA for SNAREs (as measured by qPCR; Fig. 4d), an enhanced high extracellular potassium response and an intact potassium-induced calcium response compared with WT islets (Fig. 4b). However, their glucose-induced calcium response was much lower (Fig. 4a). Although we haven’t explored this in detail, the reduced mRNA for *Slc2a2* (which encodes glucose transporter 2 [GLUT2]; Fig. 4d) suggests that beta cells at stage 2 are still secretory competent but are defective in glucose sensing.

### Granule fusion behaviour in stage 1 prediabetic islets

**Fig. 5:**
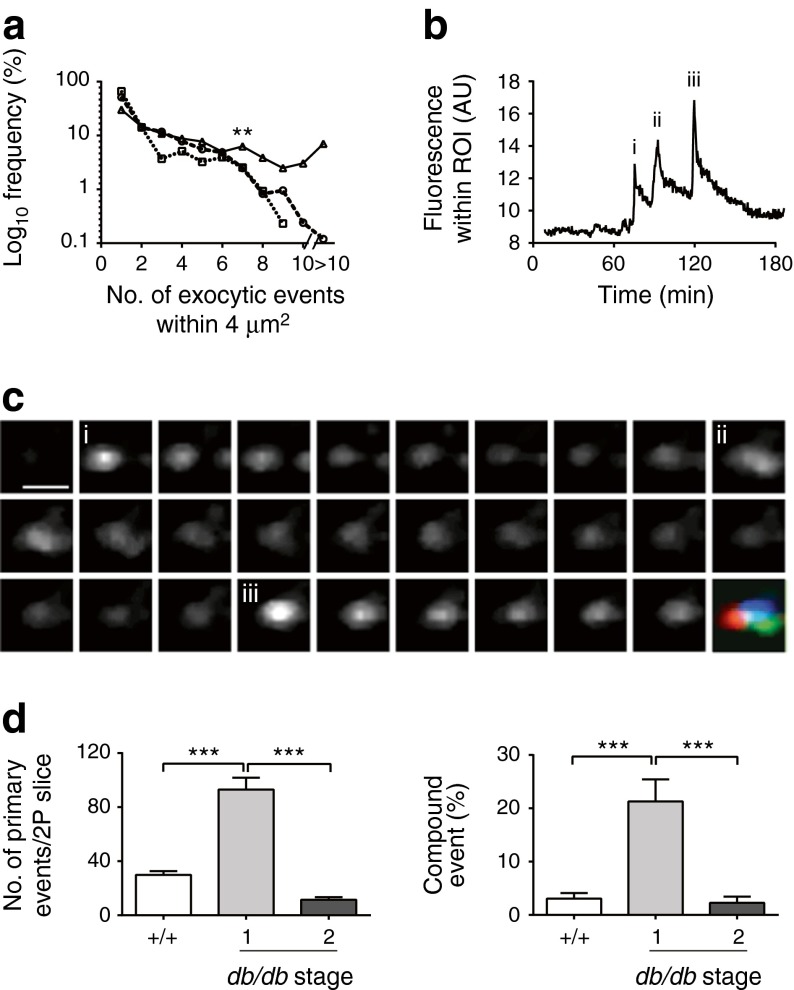
Compound exocytosis is increased in stage 1 *db/db* beta cells. (**a**) Analysis of a 4 μm^2^ region around each exocytic event shows that the frequency of observing more than seven events is much lower in WT (dashed line, circles) and stage 2 *db/db* (dotted line, squares) islets but not stage 1 *db/db* islets (solid line, triangles). (**b**) Evidence for multiple granule fusion events occurring within the same region of interest. (**c**) This is shown as an image series with the numbers (i), (ii), and (iii) indicating the peaks of fluorescence as each granule fuses. Scale bar 1 μm. (**d**) Histograms of primary (under seven events within a 4 μm^2^ region) exocytosis and compound exocytosis show increases in stage 1 and decreases in stage 2 *db*/*db* compared with WT (+/+) beta cells. **p < 0.01, ***p < 0.001. 2P, two-photon; AU, arbitrary units; ROI, region of interest

Our findings in stage 1 islets demonstrate increased insulin granule fusion. However, we also noticed a difference in the spatial pattern of granule fusion, with evidence for spatial clustering of fusion sites that was not seen in WT (see Fig. 3a). To quantify this we measured the spatial coordinates of all fusion events. We then looked at a 4 μm^2^ area around each individual fusion event and determined the frequency with which one or more other fusion events had occurred. In WT (and stage 2) islets, we observed that the frequency of other fusion events occurring around each pre-existing fusion event became significantly lower as the number of fusion events within this area increased (Fig. 5a; 27 WT and 46 *db/db* islets; 845 WT vs 1,514 *db/db* fusion granules). This suggests that, at least on this small scale, there is little evidence for the clustering of insulin granule fusion events in WT islets. In contrast, in stage 1 *db/db* islets, the frequency of multiple events (even up to ten events) remained high (Fig. 5a; 4.62% of events had seven or more fusion events within 4 μm^2^ in WT vs 22.71% in stage 1 *db/db* islets). A closer study (which was limited by our optical spatial resolution) showed that repeated fusion had occurred within the same region of interest (Fig. 5b). In many cases, this led to a build-up of fused granules suggesting sequential, compound exocytosis in which granules fuse with each other (Fig. 5c). To quantify this behaviour, we chose an arbitrary threshold of at least seven granules within a 4 μm^2^ area as suggestive of compound exocytosis and of less than seven granules as suggestive of primary exocytosis. Both the total number of primary exocytic events and the proportion of compound exocytic events increased significantly in stage 1 *db/db* islets and decreased to below WT in stage 2 *db/db* islets (Fig. 5d).

**Fig. 6:**
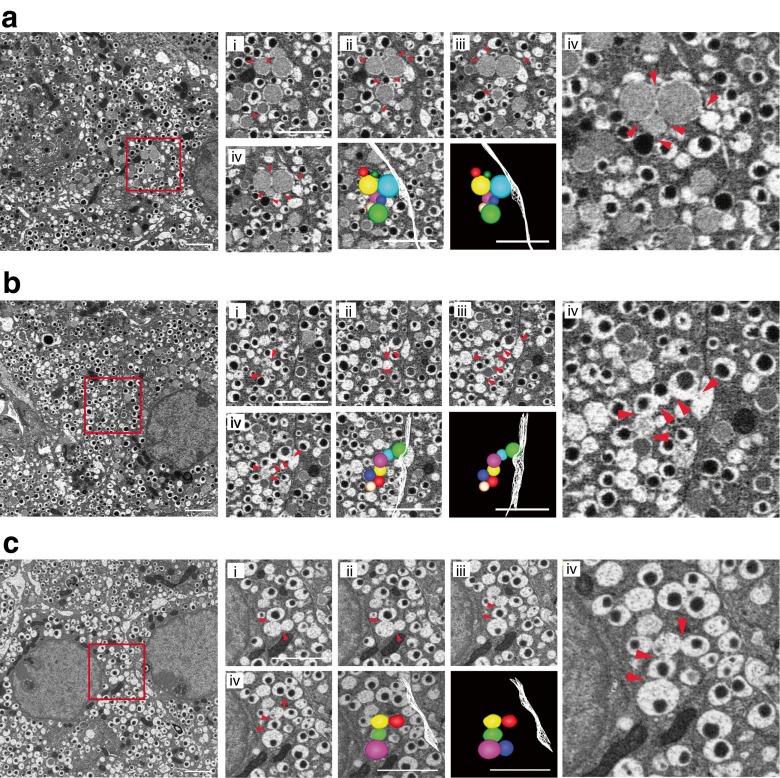
Electron microscopy evidence for compound exocytosis. (**a**, **b**) Serial block-face electron micrographs through two typical examples of multigranular compound exocytosis in *db/db* islets stimulated with 15 mmol/l glucose. Low magnification images show the area that is enlarged and shown as serial sections (i–iv) 50 nm apart taken through a compound exocytic event composed of multiple fused granules. The reconstructed models show the spatial relationships between each fused granule (in colour) and also identify the adjacent cell plasma membrane (white mesh). (**c**) In some cases, granules within the cell were fused together but made no contact with the cell surface, as shown in the model. Scale bar 2 μm

Our data is consistent with, but does not prove, compound exocytosis. We therefore undertook serial block-face electron microscopy of islets that had been fixed after 15 mmol/l glucose stimulation for 7 min (Fig. 6a, b and ESM Video 1). We could readily identify chains of fused insulin granules with a single exit point (i.e. fusion pore) at the cell membrane, thus providing good evidence for granule–granule fusion. Interestingly, we also found evidence of granule–granule fusion without contact with the cell membrane (Fig. 6c). Chains of fused insulin granules were found in both WT and *db/db* islets and could be part of the granule maturation process. Our evidence for granule–granule fusion is dependent on the appearance of continuity and the lack of a membrane between granules. Further work will be required to show granule–granule fusion and content mixing. However, and consistent with previous findings [21], analysis of the live-cell data (Fig. 5) showed that although most granule fusion events are similar in size (see Fig. 5b), some are larger and therefore could arise from multigranular objects that fuse with the cell membrane (ESM Fig. 1 [21]). We conclude that live-cell imaging and electron microscopy indicate that at stage 1 the prediabetic phenotype shows an increased prevalence of compound exocytosis.

## Discussion

We used GTTs to classify disease severity in *db/db* animals and demonstrated that changes in beta cell function do occur in prediabetes. In stage 1 *db/db* mice, the GTT is the same but FBG is elevated compared with WT mice; however, we observe an increase in islet size and insulin content, which supports previous work demonstrating beta cell expansion in early disease. Our new finding is that function is also altered, including an increase in basal insulin secretion and an increase in the number of glucose-induced granule fusion events, the latter due to upregulation of compound exocytosis.

### Beta cell function in prediabetes

Our evidence demonstrates significant changes in the secretory function of islets in prediabetes.

First, we observed an increase in basal (i.e. at 3 mmol/l glucose) insulin secretion. This increase is maintained in stage 2 islets, consistent with previous work on the *db/db* model [20, 22, 23]. The increased secretion could be driven by the elevated basal intracellular calcium concentration that was previously observed at stage 2 [20] and which we now show also occurs in stage 1 islets (Fig. 4a), possibly through calcium-dependent granule mobilisation step [24].

Second, after stimulation, we showed that stage 1 islets have more glucose-induced insulin granule fusion events compared with WT islets (Fig. 3a), but with no change in the calcium response (Fig. 4a). Since the high potassium-induced response is also bigger, again with no increase in the calcium response, these data are consistent with upregulation of the terminal stages of the stimulus secretion pathway that are downstream of calcium entry. This conclusion is supported by the increase in mRNA expression of some of the SNAREs (Fig. 4d).

A direct comparison of insulin secretion from islets (Figs 1c, 2f) shows that the fold increase in secretion is decreased at all disease stages in *db/db* islets. This largely reflects the reduced capacity of the beta cells to respond to glucose at stages 2–4. However, at stage 1 this is mostly due to increased basal secretion: glucose-stimulated secretion increases when measured per islet (Fig. 1c) but remains the same when measured per insulin content (Fig. 2f).

Given the similarities in islet insulin secretion between WT and stage 1 *db/db* islets, the large increase in insulin granule fusion numbers was surprising: the explanation for the discrepancy between increased fusion events but comparable secretory output is not clear. One possibility is heterogeneity in the beta cell content and function, which has been observed in late-stage disease [20], possibly due to cell dedifferentiation [25]. Heterogeneity might give rise to some cells that contain insulin but are non-responsive, therefore explaining the increase in granule fusion events in the other cells. An alternative possibility is that insulin granule fusion at stage 1, in particular via compound exocytosis, is less efficient. This idea is difficult to test, but we might expect that multiple granules releasing content through a common, single, fusion pore would at least have slower kinetics of insulin release compared with normal fusion, in which each granule releases its content through its own fusion pore.

Compound exocytosis is rarely observed in WT cells after glucose stimulation but is specifically upregulated, for example with glucagon-like peptide 1 (GLP-1) stimulation [26]. Although it is possible that stage 1 *db/db* islets selectively enhance pathways that normally regulate compound exocytosis, we suggest that this is more likely to result from non-specific upregulation of the exocytic machinery. Given the critical importance of the balanced expression of SNARES for regulating granule fusion [27], it is possible that an imbalance might lead to an increased prevalence of compound exocytosis.

### Comparison with disease progression in humans

Our phenotypic data showing disease progression in *db/db* mice are consistent with previous work [28] and with the progression of type 2 diabetes in humans [3]. In humans, compensatory increases in the insulin response occur in stage 1 disease [18, 29] and are associated with increased islet size and insulin content [8, 9]. As the disease progresses, insulin content decreases [30] and, as we observed in the mouse, glucose-induced insulin secretion is reduced [31, 32].

Humans with relatively poorer insulin sensitivity tend to have a higher insulin secretion [33]. The product of insulin sensitivity and pancreatic responsivity (the disposition index), has a hyperbolic relationship [33] that does not normally change over time. However, evidence, from obese humans that are developing insulin resistance, shows that insulin secretion can increase according to the demand [34]. Another human study showed increasing insulin secretion up to a maximum at a FBG of 5.6 mmol/l, followed by a decline [29]. Longitudinal studies show that individuals who progressed to diabetes did not have compensatory increases in insulin secretion; in contrast, those that did, did not progress [18, 35]. Consistent with this, animal models of diabetes also show increased insulin secretion in early disease [11, 36, 37].

Our data represent a step towards understanding the mechanisms that underlie increased insulin secretion in prediabetes and show that, in addition to an expansion in cell numbers, functional changes are occurring in the beta cell.

### Summary of disease progression to diabetes

**Fig. 7:**
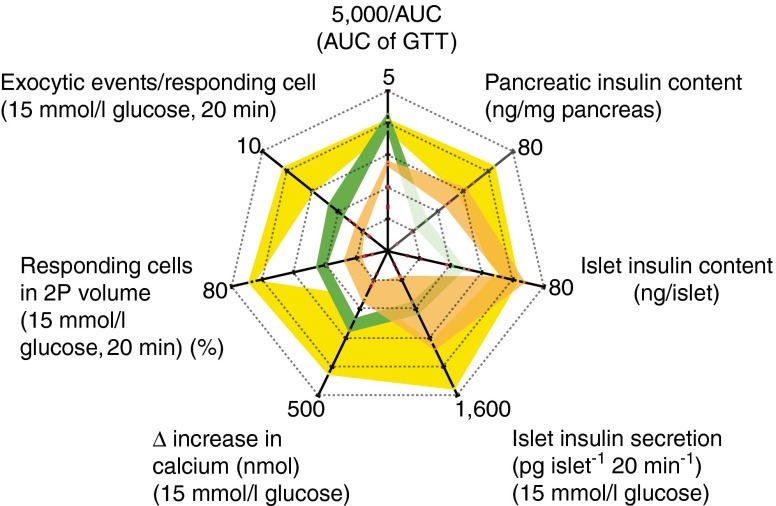
Spider graph showing changes in *db/db* islet and beta cell secretion at different stages of disease. Each line is centred on the mean for each variable and the width represents the SEM. Note, for clarity, the AUC is inverted and shown as a fraction of 5,000 mmol l^−1^ × min. Green, WT; yellow, *db/db* stage 1; orange, *db/db* stage 2. 2P, two-photon

The changes in phenotype and function associated with WT and stage 1 and 2 *db/db* animals is shown in a spider plot (Fig. 7). At stage 2, the dramatic drop in the number of granule fusion events (Fig. 3) is coincident with the increase in AUC, thus closely aligning beta cell dysfunction with a loss of glucose homeostasis. These stage 2 beta cells still secrete insulin in response to high extracellular potassium (Fig. 4b), which is consistent with effectiveness of sulfonylureas (potassium channel blockers) for treating diabetes [38]. The possibility of an upstream defect is supported by the reduced calcium response to glucose that we and others have observed [20, 23] and the decrease in *Slc2a2* mRNA levels (Fig. 4d [39]). In type 2 diabetic human islets, the loss of glucose-induced secretion, but with little change in arginine-induced insulin secretion, also suggests defects in glucose sensing [32]. In addition to these upstream defects, the altered expression of exocytic machinery that we observed is consistent with evidence that genetic variation affecting downstream factors, such as granule docking and decreased calcium sensitivity, are involved in the development of type 2 diabetes [40]. Thus, later disease is probably due to a combination of aberrant exocytosis and changes in glucose sensing.

### Conclusion

We conclude that altered beta cell function is intimately associated with prediabetes and progression to diabetes.

## Electronic supplementary material

Below is the link to the electronic supplementary material.

ESM Fig. 1 (PDF 28 kb)

ESM Methods (PDF 90 kb)

ESM Video 1 Serial electron microscopy images of a *db/db* islet stimulated with 15 mmol/l glucose showing an example of granule-to-granule fusion. Electron microscopy sections are separated by 50 nm and each fused granule was traced in the program IMOD to produce the coloured model of the granules and the plasma membrane. (AVI 3,696 kb)

## Abbreviations

FBG: Fasting blood glucose
GTT: Glucose tolerance test
IFG: Impaired fasting glucose
IGT: Impaired glucose tolerance
qPCR: Quantitative real-time PCR
SNARE: Soluble *N*-ethylmaleimide-sensitive factor attachment protein receptor
SRB: Sulforhodamine B
WT: Wild-type
