# Supplementary material for: Changes in beta cell function occur in prediabetes and early disease in the Leprdb mouse model of diabetes
Source: Diabetologia. 2016 Apr 5;59:1222–30. doi: 10.1007/s00125-016-3942-3 (PMC4869737; doi:10.1007/s00125-016-3942-3)
Supplement: Supplementary file 1 — (PDF 28 kb) [file 125_2016_3942_MOESM1_ESM.pdf]

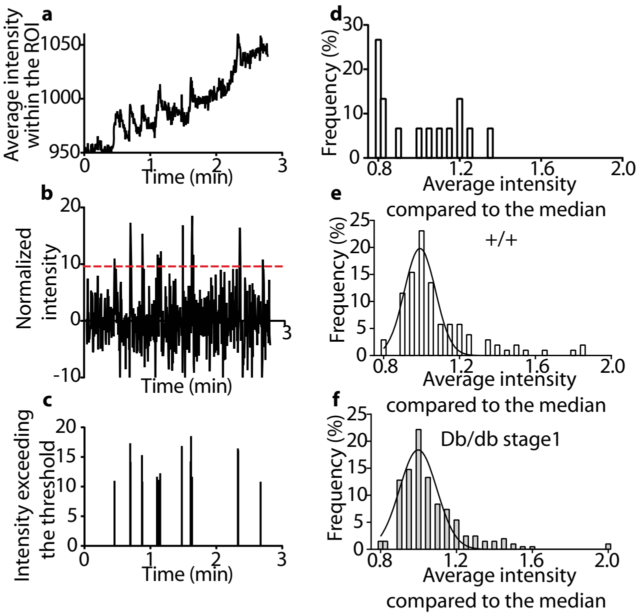

ESM Fig. 1 Automated analysis of single granule fusion event fluorescence amplitudes

(a) We randomly chose two to three 9 µm^2 regions of interest from each two-photon recording and the records, of exocytic events, such as shown in Figs. 3 and 5, were temporally filtered to produce a "baseline trace".

(b) This was then subtracted from the original to remove the fluorescence drift. To identify the time point and peak amplitude of the exocytic events we used a derivative analysis with a running subtraction of the average fluorescence signal of 10 prior time points. A threshold of 3-4 times the standard deviation of the baseline signal (shown as a dashed line on the plot) was then used to identify the peaks, as shown in (c).

(d) For each recording the median value of the peak amplitudes was used to normalise the data which, for this dataset was then used to normalise all the peak amplitudes in a frequency-amplitude plot (a value of 1 is therefore the median).

(e) Finally all data were collated across all experiments. The frequency-amplitude plot shows a peak at the median value (1) as expected. There is evidence, in +/+ and *db/db*, for larger average intensities with at around 1.2 and 1.4 of the median amplitude which supports the idea that some of the fusion events arise from granules that have prefused within the cell prior to fusion with the cell membrane.
