# Supplementary material for: Changes in beta cell function occur in prediabetes and early disease in the Leprdb mouse model of diabetes
Source: Diabetologia. 2016 Apr 5;59:1222–30. doi: 10.1007/s00125-016-3942-3 (PMC4869737; doi:10.1007/s00125-016-3942-3)
Supplement: Supplementary file 2 — (PDF 90 kb) [file 125_2016_3942_MOESM2_ESM.pdf]

Supplemental Information

Methods

Glucose tolerance test After an overnight fast (5pm- 9am), fasting blood glucose were obtained by the Accu-check Active (Roche) glucometer with a small drop of blood (~5 µl) from the tail tip. Then glucose 25% was injected intra-peritoneally (1 g/kg) and blood samples taken at 15, 30, 60, 90 and 120 min and measured for glucose concentration.

Cell preparation Isolated mouse pancreatic tissue was prepared by a liberase:collagenase, 4:1,mix (collagenase type IV, Gibco-Life Technologies, Victoria, Australia; Liberase Roche Life Sciences, Dee Why, New South Wales, Australia) digestion method in Hanks buffer (Sigma-Aldrich, NSW, Australia), adjusted to pH 7.4 with NaOH. Isolated islets were maintained (37°C, 95/5% air/CO_2) in RPMI-1640 culture medium (Gibco, Victoria, Australia) supplemented with 10% FBS (Gibco, Victoria, Australia), and 100 U/ml penicillin/0.1mg/ml Streptomycin (Invitrogen, Victoria, Australia).

Experimental Solution Experiments were performed in sodium-rich extracellular solution (in mmol/l: 140NaCl, 5KCl, 1$MgCl_2$, 2.5$CaCl_2$, 5$NaHCO_3$, 5HEPES, glucose) adjusted to pH 7.4 with NaOH.

Two-photon imaging We used a custom-made, video-rate, 2-photon microscope with a 60x oil immersion objective (NA 1.42, Olympus), providing an axial resolution (full width, half maximum) of ~1 µm [41]. Microscope peripherals (shutter, scanners etc) were controlled and images acquired, using ScanImage software.

Images (resolution of 10 pixels/µm) were analysed with the Metamorph program (Molecular Devices Corporation, Sunnyvale, CA, USA). Exocytic event kinetics were measured from regions of interest (0.78 μm^2, 78 pixels) centred over individual granules.

This indirect assay has advantages. It is a sensitive assay capable of recording single exocytic events at a rate of 4 frames per second. It spatially and temporally distinguishes exocytic events within single cells within an intact islet. It does not involve labelling of heterologously expressed proteins or loading of tracers into the cell. Past work, studying fusion timecourse, kinetics and quantity, has shown it is a faithful measure of insulin granule exocytosis.

Insulin assay To acquire insulin samples, single pancreatic mouse islets were transferred from culture to a petri dish containing a sodium-rich extracellular solution (3 mmol/l glucose) and incubated (37°C, 95/5% air/CO_2) for 30 minutes. Thereafter, groups of three islets were transferred to individual eppendorf tubes containing the sodium-rich extracellular solution, 0.2% bovine serum albumin and a stimulus such as glucose (3 mmol/l/ 15mmol/l) or high potassium (50 mmol/l) Islets were then incubated (37°C, 95/5% air/CO_2) for 20 minutes. Supernatant from each tube was collected and used for HTRF as per Cisbio insulin kit instructions. Briefly, for each sample, 10 µl of islet supernatant, 5 µl of anti-insulin Europium cryptate and 5 µl of anti-insulin XL665 were sequentially added to a 384 well plate (Perkin Elmer Proxiplate 384-Plus, cat. no. 6008280). The plate was incubated on lab bench overnight then read on a Tecan Infinite 200PRO (Tecan, Port Melbourne, Vic, Australia) machine. Insulin concentrations (ng/ml) for each well were determined from an insulin standard curve.

Calcium measurement After 2 days in culture, islets were loaded with 4 µm/l Fura-2-AM (Invitrogen, Victoria, Australia), 0.02% pluronic acid (Sigma Aldrich) and incubated (37^0C, 95/5% air/CO_2) for 30 minutes. After labelling, the islets were washed and incubated in a Na-rich extracellular buffer containing 3 mmol/l glucose for an additional 5 minutes. Then single islets were transferred to a small volume chamber mounted on the stage of an inverted fluorescence microscope with a 25x objective. The cells were alternatingly excited at 340 and 380 nm, and the ratio was collected at 490 nm.

Immunofluorescence. Islets were washed in warmed PBS, fixed in 4% paraformaldehyde/ for 15 minutes and blocked for 1 hour in 3% donkey serum, 3% bovine serum albumin and 0.3% TritonX. Islets were then incubated in primary antibodies, rabbit anti-glucagon (Sigma Aldrich, 1:200) and guinea pig anti-insulin (Abcam, 1:200) overnight at 4^0C, washed 3 times and then incubated in secondary antibodies Alexa Fluor 546 donkey anti-rabbit and Alexa Fluor 633 goat anti-guinea pig (Invitrogen, 1:200) for 3 hours. DAPI (Sigma Aldrich, Australia, 100 ng/ml final concentration) was used. Images were obtained on an Olympus FV1000 confocal microscope with a 63 X oil, 1.4 NA objective.

qPCR. Complementary RNA was removed by incubation at 37°C for 20 minutes with 2U of RNase H. Real-time PCR was carried by the Applied Biosystems, Quantstudio RT-PCR with samples holding at 50°C for 2 minutes, 95°C for 10 minutes, and 40 cycles at 95°C for 15 seconds and 60°C for 1 minute. In a 20 µl reaction volume, 10 µl SYBR Green 2x master mix (Invitrogene) was applied with the cDNA and optimized primer condition (200 nM). A dissociation curve analysis was applied immediately after the real-time cycles to confirm reaction specificity. Results were analysed according to the arithmetic formula of the comparative Ct method (Fold difference = 2−ΔΔCt, ΔΔCt = ΔCt test sample – ΔCt calibrator sample). 18S was used as the endogenous control. mRNA level of genes of interest are presented as the fold difference between the *db/db* and WT islets. Predesigned primers (Sigma Aldrich, Australia) were used as follows: *Snap25* forward, GGACTTTGGTTATGTTGGATG; *Snap25* reverse, GGATTTAAGCTTGTTACAGGG; *Stx1a* forward, AGATGATTGACAGGATCGAG; *Stx1a* reverse, GTGGTTTCTATCCAAAGATGC; *Vamp2* forward, ATCATCGTTTACTTCAGCAC; *Vamp2* reverse, TGAAAGATATGGCTGAGAGG; *Slc2a2* forward, 5’-TCAGAAGACAAGATCACCGGA-3’; *Slc2a2* reverse, 5’-GCTGGTGTGACTGTAAGTGGG-3’.

Serial block-face scanning electron microscopy Fixed islets were double post-fixed by using 2% OsO_4 with 1.5% potassium ferricyanide followed by 1% thiocarbohydrazide and then another 2% OsO_4. Samples were stained overnight with 1% uranyl acetate and then for 1 hour at 60^oC in Walton’s lead aspartate. They were then serially dehydrated with acetone and then embedded with Durcapan resin and polymerised. Individual islets were cut out of the resin, mounted and then imaged and sectioned using a Zeiss Sigma scanning electron microscope fitted with a 3View (Gatan, CA, USA) at 2.25kv and 10Pa. The resultant images were analysed using the programme IMOD [42] and 3D reconstructions performed.
